# Molecular subtyping of ependymoma and prognostic impact of Ki-67

**DOI:** 10.1007/s10014-021-00417-y

**Published:** 2021-11-23

**Authors:** Ka Young Lim, Kwanghoon Lee, Yumi Shim, Jin Woo Park, Hyunhee Kim, Jeongwan Kang, Jae Kyung Won, Seung-Ki Kim, Ji Hoon Phi, Chul-Kee Park, Chun-Kee Chung, Hongseok Yun, Sung-Hye Park

**Affiliations:** 1 Department of Pathology, Seoul National University College of Medicine, 101 Daehak-ro, Jongno-gu, Seoul, 03080 Republic of Korea; 2 Department of Neurosurgery, Seoul National University College of Medicine, 101 Daehak-ro, Jongno-gu, Seoul, 03080 Republic of Korea; 3 Department of Precision Medicine, Seoul National University College of Medicine, 101 Daehak-ro, Jongno-gu, Seoul, 03080, Republic of Korea; 4 Institute of Neuroscience, Seoul National University College of Medicine, 101 Daehak-ro, Jongo-gu, Seoul, 03080 Republic of Korea

**Keywords:** Ependymoma, Cancer genetics, Epigenetics, Prognostic factor, Gene copy number, Meta-analysis

## Abstract

Although ependymomas (EPNs) have similar histopathology, they are heterogeneous tumors with diverse immunophenotypes, genetics, epigenetics, and different clinical behavior according to anatomical locations. We reclassified 141 primary EPNs from a single institute with immunohistochemistry (IHC) and next-generation sequencing (NGS). Supratentorial (ST), posterior fossa (PF), and spinal (SP) EPNs comprised 12%, 41%, and 47% of our cohort, respectively. Fusion genes were found only in ST-EPNs except for one SP-EPN with *ZFTA-YAP1* fusion, NF2 gene alterations were found in SP-EPNs, but no driver gene was present in PF-EPNs. Surrogate IHC markers revealed high concordance rates between L1CAM and *ZFTA*-fusion and H3K27me3 loss or EZHIP overexpression was used for PFA-EPNs. The 7% cut-off of Ki-67 was sufficient to classify EPNs into two-tiered grades at all anatomical locations. Multivariate analysis also delineated that a Ki-67 index was the only independent prognostic factor in both overall and progression-free survivals. The gain of chromosome 1q and CDKN2A/2B deletion were associated with poor outcomes, such as multiple recurrences or extracranial metastases. In this study, we propose a cost-effective schematic diagnostic flow of EPNs by the anatomical location, three biomarkers (L1CAM, H3K27me3, and EZHIP), and a cut-off of a 7% Ki-67 labeling index.

**Supplementary Information:**

The online version contains supplementary material available at 10.1007/s10014-021-00417-y.

## Introduction

Ependymomas (EPNs) are uncommon neuroepithelial malignancies, constituting approximately 2% of the central nervous system (CNS) tumors and about 6.8% of all gliomas [1]. They can occur at any age and are the third most common CNS tumors in children [2]. 2016 World Health Organization (WHO) classification subdivided EPNs mostly based on histology and included only one genetically defined EPN subtype, EPN, *RELA* fusion-positive [3], and it has been noted that the WHO grades do not reflect biological behavior and patient outcomes [4]. *RELA* fusion-positive EPN has been revised into *ZFTA* fusion-positive in the 2021 WHO classification [5].

The cIMPACT-NOW update 7 and the 2021 WHO classification of tumors of the CNS classify EPNs by a combination of anatomic site, molecular genetics, epigenetics, and histological features [6, 7]. According to these updates, anatomic sites, e.g., ST, PF, and SP, should add to the diagnostic term, EPN. However, the criteria for the grades are not clearly defined in the WHO classification. In general, grade 3 EPN is an infiltrative, highly proliferative tumor with active mitosis and microvascular proliferation, but necrosis and nuclear polymorphisms may be present in both grades of EPN and are, therefore, not the parameters of EPN grade. As we already know, nuclear polymorphism itself is very rare in both grades of EPNs. Also, the mitotic rate is not precisely defined. In 2016 WHO blue book mentioned that the interpretation of the most histopathological variables in EPN grading is subjective, and multivariate analyses of prognostic variables are limited [8]. Therefore, histology- or molecularly defined EPN grades still have pitfalls as there are no clear criteria established for WHO grades 2 and 3.

*ZFTA (zinc 119 finger translocation associated*, previous term, *c11orf95*) fusion-positive ST-EPN is considered a grade 3 associated with poor outcomes. *YAP1 (yes associated protein 1*) fusion-positive ST-EPN [6, 7, 9] is associated with a good prognosis and grade 2. For the past 5 years, neuropathologists have been applying 2016 WHO classification to the diagnosis of CNS tumors; thus, it is necessary to add EPN-*RELA* fusion-positive to ST-EPN-*ZFTA* fusion-positive. PF-EPN is characterized by the absence of somatic mutation of genes. There are two groups of PF-EPN; group A (PFA) and group B (PFB). PFA-EPN commonly occurs in children, exhibits aggressive behavior, is considered to be of grade 3, and is characterized by loss of H3K27me3 and overexpression of Zest 2 Polycom Inhibited Complex (EZHIP) inhibitory proteins [10, 11]. EPNs of this subtype show increased CpG methylation at CpG islands, demonstrating a CpG island methylator phenotype (PFA-CIMP^+^) [11]. PFB-EPN has no genetic or epigenetic abnormalities, mostly in adults, and is classified as grade 2 because of a good prognosis. In most SP-EPNs, the *NF2* gene is inactivated by mutation or deletion and the new subtype with *MYCN* amplification is associated with a dismal prognosis [12].

However, EPNs grading based on histological parameters is required when molecular studies fail to reveal reliable results or when a molecular genetic study is unavailable. In addition, some exceptional cases of grade mismatch with molecular genetic subtypes such as WHO grade 2 PFA or grade 3 PFB, and grade 2 *ZFTA-RELA* fusion-positive have been reported [4, 7, 13]. cIMPACT-NOW update 7 explained that although molecular properties are closely related to prognosis, molecularly defined EPN grades are not mature enough to grade EPNs [7]. In addition, the histological grade is still important because the 5-year overall survival (OS) of grade 3 SP-EPN in a recent large cohort was significantly worse than that of grade 2 SP-EPN [14].

A Ki-67 index greater than 5%, 7%, or 10% has been reported to be significantly associated with poor prognosis [15–18]. It is also important to determine the treatment options after surgery. However, there has been no consensus on the cut-off in previously published studies.

Here, we reclassified our series of EPNs for 20 years by anatomical site, genetic, and epigenetic classifiers according to the cIMPACT-NOW update 7 and 2021 WHO classification [6, 7]. We also studied the association of copy number variation (CNV) with clinical outcomes. We propose a cost-effective schematic diagnostic flow, easy to apply in clinical and pathological practice with a 7% cut-off for the Ki-67 index.

## Materials and methods

### Patient population

The clinicopathological features of 223 biopsy-proven ependymomas were reviewed, which were obtained from the archives of Seoul National University Hospital (SNUH) between 2000 and 2020. Eight two EPNs were excluded, which were myxopapillary EPNs (*n* = 19), subependymoma (*n* = 2), recurrent EPNs (*n* = 30), missing paraffin blocks (*n* = 17), suboptimal IHC results by poor block quality (*n* = 12), and diagnosis revision (*n* = 2).

**Fig. 1:**
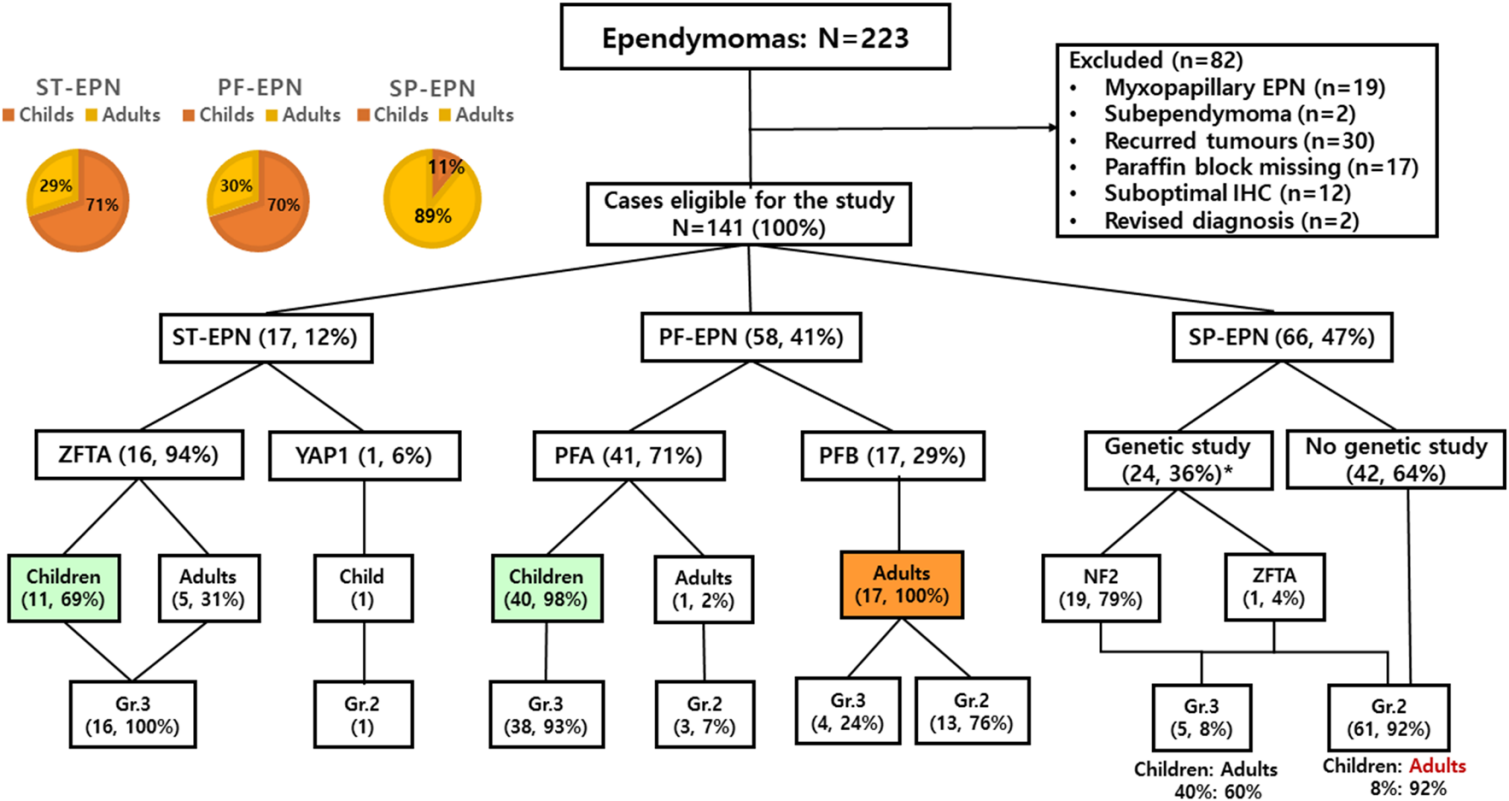
The patients’ population in the study. There were a total of 141 EPNs: 17 (12%) ST-EPNs, 58 (41%) PF-EPNs, and 66 (47%) SP-EPNs. This schematic view is the result of the classification of our EPNs according to the cIMPACT-NOW update 7

IHC and molecular studies successfully reclassified 141 primary EPNs, including 17 (12%) ST-EPNs, 58 (41%) PF-EPNs, and 66 (47%) SP-EPNs according to the cIMACT-NOW update 7 and the 2021 WHO classification (Fig. 1) [5]. Seventy percent of ST-EPNs-*ZFTA* fusion-positive (Child: 1– to 16-year-old, median: 8-year-old; Adult: 32–51, Median: 41-year-old) and 1 case of ST-EPNs *YAP1-MAMLD1* fusion-positive occurred in children (Table 1). Interestingly, all PFA-EPNs occurred in children (0– to 16-year-old, median: 2.8-year-old), except for one clear cell PFA-EPN, occurring in a 38-year-old woman, while 100% of PFB-EPNs and 89% of SP-EPNs occurred in adults.

**Table 1:** Epidemiology of our patients with ST-, PF- and SP-EPNs

Subtype		ST-EPN-*ZFTA* (*n* = 16)	ST-EPN-*YAP1* (*n* = 1)		PFA (*n* = 41)	PFB (*n* = 17)		SP-EPN-Grade 2 (*n* = 61)	SP-EPN-Grade 3 (*n* = 5)
Gender	M: F	5: 3	F (1)		1.6: 1	1: 1.4		1.2: 1	1:4
Age (years)	Total: median (range)	14 (1–51)	6		3 (0–16)	41 (21–69)		49 (8–73)	26 (5–49)
	Children	8 (1–16)							
	Adult	41 (32–51)							
Site	Frontal lobe	11*	1	4th V	17	13	C	37	1
	Parietal lobe	4*	–	4th V + 3rd V	1	0	C & T	8	0
	Occipital lobe	1	–	4th V + CPA	1	0	T	11	2
	Temporal lobe	1*	–	Brainstem	2	0	T & L	2	0
	Basal ganglia	2*	–	CMJ	0	1	L	3	2
	Thalamus	2*	–	Cerebellum	10	1			
	Lateral V	1	–	CPA	10	2			
Treatment	GTR only	1	1		4	4		59	1
	GTR + RT	7	–		7	3		–	3
	GTR + CT	1	–					–	
	GTR + CCRT	3	–		12	2		1	1
	STR + RT	1	–		4	5		1	–
	STR + CCRT	3	–		13	2		–	–
	Data missed		–		1	1		–	–
F–U (months)	Median (range)	42 (3–99)	99		48 (1–234)	49 (0–139)		37 (0–1003)	8 (0–39)
	No. of dead (%)	3 (19%)	–		15 (37%)	1 (6%)		4 (7%)	0 (0%)
	No. of recur (%)	9 (56%)	–		29 (71%)	4 (24%)		5 (8%)	1 (20%)
Survival (months)	OS: median (range)	42 (3–100)	99		49 (5–234)	55 (0–139)		39 (0–1003)	8 (0–39)
	PFS: median (range)	23 (0–65)	99		19 (0–216)	48 (0–139)		38 (0–1003)	8 (0–39)

*V* ventricle, *GTR* gross total resection, *STR* subtotal resection, *RT* radiation therapy, *CT* chemotherapy, *CCRT* concurrent CT and RT, *CPA* cerebello-pontine Angle, *CMJ* cervicomedullary junction, *ST* supratentorial, *C* cervical spinal cords, *T* thoracic spinal cords, *Ls* lumbar spinal cords, *F/U* follow-up Median (range)

*In the cases of multiple sites, they were counted twice

Informed consent for the genetic study of EPNs was obtained from the patients or parents of the children. The institutional review board of our hospital approved this study (Approval No. 2012-034-1179), which was conducted under the ethical standards outlined in the 1964 Declaration of Helsinki and subsequent amendments.

### Pathology and immunohistochemistry (IHC)

All tumors were reviewed by two pathologists (KY Lim and SH Park). Histological features including histologic subtype, mitotic rate, necrosis, and microvascular proliferation were evaluated. IHC staining was performed on 3-µm-thick formalin-fixed and paraffin-embedded (FFPE) tissues using an automated immunostaining system (BenchMark ULTRA system; Ventana-Roche, Mannheim, Germany). The primary antibodies used in this study are summarized in Supplementary Table 1.

The Ki-67 labeling indices were calculated the positive nuclei per 500 tumor cells by SpectrumPlus Aperio morphometric algorithm in the several hot spots, and the highest index was determined. The mitotic rate was counted in 10 HPFs of the hot spot area with pHH3 IHC.

IHC readings were semi-quantitatively performed in intensity and distribution. Null, ambiguous, weak, moderate, and strongly positive were scored as 0, 1, 2, 3, and 4, respectively. In our cases, H3K27me3 loss was completely negative in tumor cells, EZHIP overexpressing cases showed diffuse strong positive, and EZHIP negative cases were either completely negative or a few positive cells.

The concordance rates of L1CAM and NF-kB with ST-EPNs were analyzed and both H3K27me3 loss and EZHIP overexpression were used for the diagnosis of PFA-EPNs. We also determined an adequate cut-off of the Ki-67 labeling index to differentiate grade 2 from grade 3, and compared it with the genetic subgroups and clinical outcomes.

### DNA and RNA extraction and genetic studies by NGS

Representative areas of the tumors with more than 90% tumor cell content were outlined on the FFPE sections for macrodissection. DNA and RNA extractions were performed from serial sections using the Maxwell^®^ RSC DNA FFPE Kit (Promega, Madison, WI), according to the manufacturer’s instructions.

NGS was performed in 60 cases of EPNs (16 ST-, 23 PF-, and 21 SP-EPNs) with the NextSeq™ 550 system (Illumina, San Diego, CA) using a customized brain tumor gene panel (FIRST Brain Tumor Panel of SNUH), which contains 207 genes including *MYCN*, and 54 fusion genes including *ZFTA, RELA*, and *YAP1.* This gene was approved by the Korean Ministry of Food and Drug Safety.

Sequencing data were analyzed using the SNUH FIRST Brain Tumor Panel Analysis pipeline. The quality control of the fastq file was performed and only the data that met the criteria were analyzed. Paired-end alignment to the hg19 reference genome was performed using BWA-mem and GATK Best Practice [19]. Second quality control was performed to ensure that additional variant calls were appropriate. The open-source tools used were GATK UnifedGenotyper, SNVer, and LoFreq for SNV/InDel detection [20], Delly and Manta for translocation discovery [21], THetA2 for purity estimation, and CNVKit for CNV calling [22–25]. SnpEf annotated the variants detected from various databases, such as RefSeq, COSMIC, dbSNP, ClinVar, OncoKB, and gnomAD. The germline variant was then filtered using the population frequency of these databases (> 0.01%). Finally, the variants were confirmed through a comprehensive review of a multidisciplinary molecular tumor board. Whole exome sequencing was carried out in 5 cases of ependymomas, which were evaluated with similar pipelines of NGS.

### Meta-analysis

To verify the prognostic effect of the Ki-67 labeling index, 11 articles published between 2000 and 2021 were reviewed [16, 18, 26–34]. Ki-67 cut-off, OS, PFS, HR, 95% CI, and *P*-value were included in the meta-analysis. In multiple studies, where cohorts overlapped, the most complete was used. Cochran’s Q test was applied to determine statistical inter-study heterogeneity: Q > 40 and *P*-value > 0.10 (not 0.05) were considered to determine the presence of inter-study heterogeneity [35].

### Statistical analysis

The survival rate of patients according to clinical, pathological, and genetic factors was analyzed using Kaplan–Meier survival analysis and log-rank test. Cox regression analysis was used in univariate and multivariate analysis to study the prognostic impacts of clinical and histopathological parameters. First, the influence of each variable was evaluated, and *P* ≤ 0.05 was interpreted as statistical significance. Second, the prognostic effect and independence were evaluated by examining only the variables with *P* ≤ 0.05 in the multivariate analysis. All statistical analyses were performed using SPSS 25 (IBM, Armonk, NY), R version 3.5.3 (R Foundation, Vienna, Austria), and survminer 0.4.6 packages.

## Results

### Epidemiology and subgroup of ST-, PF- and SP-EPN

IHC and molecular studies successfully reclassified 141 primary EPNs, including 17 (12%) ST-EPNs, 58 (41%) PF-EPNs, and 66 (47%) SP-EPNs (Fig. 1, Table 1). ST-EPNs-*ZFTA* and ST-EPN-*YAP1* were 94% and 6% out of 17 ST-EPNs. The frontal lobe was the most commonly affected area. The median age of 16 ST*-*EPN-*ZFTA* patients was 14 years (range 1–51 years, 44%: younger than 10 years). All were *ZFTA-RELA* fusion except for one *ZFTA-MAML2* fusion.

PFA and PFB consisted of 71% and 29% of 58 PF-EPNs. Overall, 70% of all PF-EPNs and 98% (40/41) of PFA-EPNs occurred in children (median 3 years, range 1–16 years). One clear-cell PFA-EPN occurred in a 38-year-old woman. One hundred percent of PFB-EPNs occurred in adults (median age 41 years, range 21–69 years). The fourth ventricle was the most commonly affected site. Most patients with PFA-EPNs received adjuvant treatments after surgical resection.

In 66 cases of SP-EPNs, grade 2 and grade 3 were 92% and 8%, respectively and 89% were adults and 11% were children. Grade 3 SP-EPNs occurred in young patients (median 26 years; range 5–49 years, child: adult = 4: 6), whereas grade 2 EPNs occurred in a broad age range of patients (median 49 years; range 8–73 years). The cervical spinal cord was the most frequently affected site by grade 2 SP-EPNs (74%, 45/61).

*NF2* alterations were observed in 79% (16/21) of SP-EPNs that had undergone NGS, with exception of three known NF2 patients. Three NF2 patients presented with multiple benign tumors, including meningiomas or schwannomas in addition to WHO grade 2 EPN. Our cohort did not have *MYCN* amplified SP-EPNs.

*NF2* copy number aberration was found in 81.5% (13/16) of NGS performed WHO grade 2 SP-EPNs (*NF2* deletion in 44%; monosomy 22 in 37.5%). The remaining three cases each had multiple CNV, monosomy 6, and a balanced chromosome without mutation.

NF2 alteration was found in 60% (3/5) of WHO grade 3 SP-EPNs; two pathogenic mutations of NF2 (splicing, c.1122 + 1G > A, and splicing, c.599 + 1G > A), and one *NF2* gene deletion. The remained one had *ZFTA-YAP1* fusion, which was just reported [36]. The other one had a balanced chromosome with no mutations (Supplementary Table 2). Thus, *NF2* status was not related to histological grade or biological behavior.

### The results of the immunohistochemical study

**Fig. 2:**
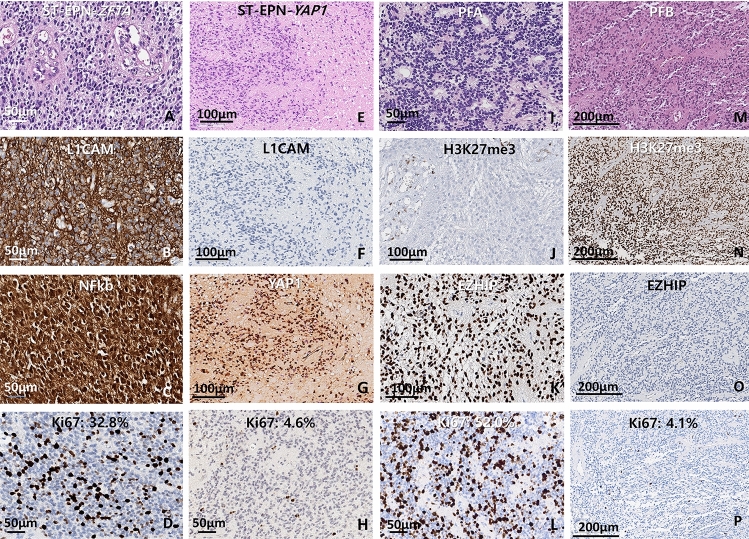
Immunohistochemistry results of the ST-EPNs (**A**–**H**) and PF-EPNs (**I**–**P**). ST-EPN-ZFTA presented prominent microvascular proliferation. IHC results show robust and diffuse cytoplasmic and membranous staining for L1CAM and nuclear staining for NFkB with a high Ki-67 index (32.8%) (**A**–**D**). ST-EPN-YAP1 presented focal necrosis without microvascular proliferation. IHC results present negativity for L1CAM and strong nucleus staining of YAP1 with low Ki-67 index (4.6%) (**E**–**H**). PFA is characterized by loss of H3K27me3 expression, EZHIP overexpression, and a high level of the Ki-67 index. Some cases show extremely high proliferative activity with over 50% of the Ki-67 index (**I**–**L**). PFB is characterized by retained expression of H3K27me3, EZHIP negativity, and low Ki-67 indices (**M**–**P**). (Length of the bar, magnificence: 50 μm, ×200 (**A**–**D**, **H**, **I**, **L**); 100 μm, ×100 (**E**–**G**, **J**, **K**); 200 μm, ×80) (**M**–**P**)

L1CAM was positive in 94% (16/17) of ST-EPN, which had *ZFTA-RELA* fusion (15 cases) and *ZFTA-MAML2* (1 case) by NGS. One case (6%) of ST-EPN-*YAP1-MAMLD1* fusion was negative for L1CAM (Fig. 2 and Supplementary Table 3).

All PFB-EPNs were negative for both H3K27me3 and EZHIP (Fig. 2). There was no case of immunoreactive for K27M (antibody for histone Lys27Met). The exceptional EZHIP-negative PFA-EPN, which was reconfirmed as EPN by electron microscopy, showed characteristic ependymal features such as intracytoplasmic and intercellular microrosettes with microvilli and cilia as well as zonula adherens, but methylation study could not be performed due to poor DNA quality. It was WHO grade 3 occurred in the fourth ventricle of an adult (38-year-old).

### Mitotic rates and Ki-67 labeling indices

**Fig. 3:**
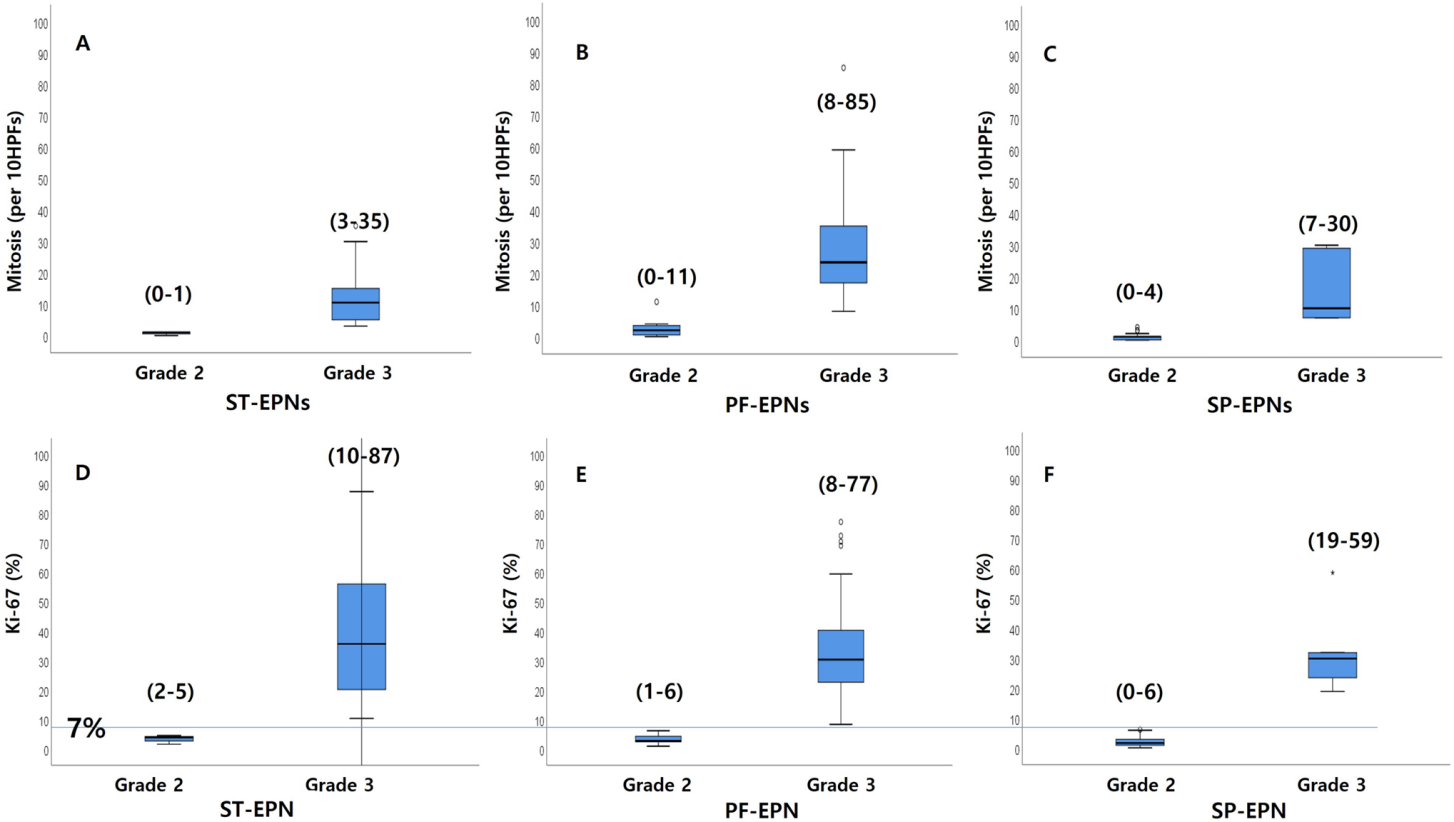
The distribution of the mitotic counts (**A**–**C**) and the Ki-67 level (**D**–**F**) of ST-, PF- and SP-EPNs are plotted. There was an overlap of mitotic rates between the grades and genetic and epigenetic groups. In contrast, there was no overlap of the Ki-67 labelling indices between the Ki-67 low- and high-groups

There was an overlap of mitotic rates between the grade and genetic and epigenetic groups (Fig. 3A–C). In contrast, there was no overlap of the Ki-67 labeling indices between Ki-67 low- and high-groups. The Ki-67 labeling index of grade 2 and grade 3 ST-EPN ranged 1.62–4.60% (median 3.1%) and 10.4–87.4% (median 35.7%), respectively. In PF-EPNs, the median labelling index of low and high Ki-67 group was 2.8% (range 0.94–6.20%, *n* = 16) and 30.4% (range 8.4–77.1%, *n* = 42). We concluded that a Ki-67 index of 7% is a good cut-off for the EPN grades and in all anatomical locations (Fig. 3D–F).

### Copy number variation in NGS-performed 60 ependymomas

CNV analysis by NGS was performed (16 ST-EPNs, 23 PF-EPNs, and 21 SP-EPNs) (Supplementary Table 2). Of the six tumors with chromosome 9p21 deletion, 84% (5/6) recurred at least twice, four tumors (67%) had extracranial metastasis, and three patients (50%) died. Seven tumors with a 1q25 gain recurred in 87.5% (7/8); four patients (50%) had two or more recurrences, of which one patient (12.5%) had extracranial metastasis, and 2 patients (25%) died.

Monosomy 6 was found in one case of ST-EPN-*ZFTA*, two PFA, and one PFB. One PFA-EPN with monosomy 6 had multiple recurrences and extracranial metastasis. Monosomy 11 was only observed in ST-EPN-*ZFTA* and there was no recurrence during the 3-year follow-up period. Chromosome 1p loss was observed in one ST-EPN-*ZFTA*, which also presented extracranial metastasis.

A balanced chromosomal profile (*n* = 12) was observed at all anatomical locations, including five ST-EPN-ZFTAs, one *YAP1-MAMLD1* fusion-positive ST-EPN, three PFA-EPNs, and three SP-EPNs. Among them, two cases showed intracranial recurrent EPN (2/9, 22%) and one case showed extracranial metastasis. Interestingly, an SP-EPN with a balanced copy number and *ZFTA-YAP1* fusion recurred 8 months after GTR which was clustered with *ZFTA-RELA* fusion-positive ST-EPN by the methylation profile [36]. Multiple CNV was observed in two PFB-EPNs with no recurrence.

### Recurrence rate and survival

All patients with ST-EPN-*ZFTA* received adjuvant treatments after the surgical resection, except one case with gross total resection (GTR) only (Table 1). Despite adjuvant treatment, 56% (9/16) of ST-EPN-*ZFTA* had one or more recurrences and three of them died (3/9), 41% (7/16) had multiple recurrences, and 12.5% (2/16) recurred more than five times. Extracranial metastases were found in 25% (4/16) of ST-EPN-*ZFTA* into the liver, lung, and salivary gland. The patient with ST-EPN *ZFTA-MAML2* fusion-positive (5-year-old girl) had recurred 49 months after GTR + RT. ST-EPN *YAP1-MAMLD1* fusion-positive (6-year-old girl) did not recur for 99 months after GTR, without adjuvant therapy. The 5-year and 10-year OS rates of ST-EPN-*ZFTA* were 93.8% and 81.3%, respectively. The 5-year and 10-year PFS rates of ST-EPN-*ZFTA* were 50.0% and 43.8%, respectively. 

The Kaplan–Meier analysis of PFS rates was significantly worse for PFA than for PFB (*P* = 0.007); 71% of PFA-EPN (29/41) and 24% (4/17) of PFB-EPNs. Multiple recurrences and extracranial metastases were observed in 39% and 24%, respectively. Among them, 44% were younger than 5 years of age. The 5-year and 10-year OS rates of PFA-EPN were 73.2% and 70.7%, respectively. The 5- and 10-year PFS rates of PFA-EPN were 36.6% and 29.3%, respectively. None of the PFB-EPNs showed extracranial metastasis.

Of the five grade 3 SP-EPNs, three received GTR + RT and two underwent GTR. All grade 2 patients had only GTR, except for two patients receiving STR + RT. OS and PFS were similar to the follow-up period for WHO grade 2 SP-EPNs (OS: median 39 months; range 0–1003 months, PFS: median 38 months; range 0–1003 months).

### Univariate and multivariate analysis

In univariate and multivariate studies in a total of 75 intracranial EPNs, the variables were age (age < 4 vs. age ≥ 4), gender, location, the extent of resection (GTR vs. STR), microvascular proliferation, necrosis, nuclear pleomorphism, mitotic index, Ki-67 labeling index and three biomarkers L1CAM, H3K27me3, and EZHIP. Only a Ki-67 index was significantly associated with OS in univariate analysis (*P* = 0.046) (Table 2A). The microvascular proliferation (*P* = 0.048), necrosis (*P* = 0.044), mitotic activity (*P* = 0.001), Ki-67 index (*P* < 0.001), H3K27me3 loss (*P* = 0.016), and EZHIP overexpression (*P* = 0.015) were significantly associated with PFS. Age and the extent of resection showed clear trends but did not reach statistical significance. In multivariate analysis, the Ki-67 index (*P* = 0.001) was the only independent prognostic factor associated with PFS (Table 2B).

**Table 2:** Univariate and multivariate analysis of clinicopathological parameters on OS and PFS in a total of 75 intracranial EPNs

(A) Overall survival				
Variables		Univariate		
		*P*	HR	95%CI
Age	age ≥ 4 vs. < 4	0.219	1.72	0.72–4.11
Sex	Male vs. Female	0.36	0.66	0.27–1.61
Location	ST vs. PF	0.97	1.02	0.36–2.87
Extent of resection	GTR vs. STR	0.99	1.003	0.41–2.44
Histologic type	Classic vs. anaplastic	0.38	0.67	0.27–1.65
Microvascular proliferation	Present vs. absent	0.79	0.89	0.37–2.11
Necrosis	Present vs. absent	0.15	0.45	0.15–1.34
Nuclear pleomorphism	Present vs. absent	0.39	0.62	0.21–1.83
Mitotic index	Continuous category	0.051	1.02	1.00–1.05
Ki-67	Continuous category	0.046	1.02	1.00–1.04
L1CAM expression	Positive vs. negative	0.94	1.05	0.30–3.70
H3K27me3 loss	Loss vs. retained expression	0.06	6.86	0.91–51.94
EZHIP overexpression	Overexpression vs. negative	0.11	0.40	0.13–1.23

(B) Progression-free survival							
Variables		Univariate			Multivariate		
		*P*	HR	95%CI	*P*	HR	95%CI
Age	age ≥ 4 vs. < 4	0.055	0.95	0.89–1.001			
Sex	Male vs. Female	0.51	0.81	0.45–1.48			
Location	ST vs. PF	0.89	1.04	0.55–1.99			
Extent of resection	GTR vs. STR	0.07	0.58	0.33–1.05			
Histologic type	Classic vs. anaplastic	0.13	0.63	0.35–1.15			
Microvascular proliferation	Present vs. absent	0.048	0.55	0.30–0.99	0.69	1.16	0.56–2.40
Necrosis	Present vs. absent	0.044	0.5	0.25–0.98	0.10	0.53	0.25–1.12
Nuclear pleomorphism	Present vs. absent	0.19	0.62	0.30–1.26			
Mitotic index	Continuous category	0.001	1.03	1.010–1.040	0.36	0.99	0.95–1.02
Ki-67	Continuous category	< 0.001	1.03	1.015–1.041	0.001	1.03	1.01–1.04
L1CAM expression	Positive vs. negative	0.62	0.83	0.39–1.75			
H3K27me3 loss	Loss vs. retained expression	0.016	2.58	1.19–5.60	0.77	1.19	0.37–3.80
EZHIP overexpression	Overexpression vs. negative	0.015	0.44	0.23–0.85	0.06	0.53	0.27–1.03

### The result of meta-analysis for identifying the prognostic effect of Ki-67

**Fig. 4:**
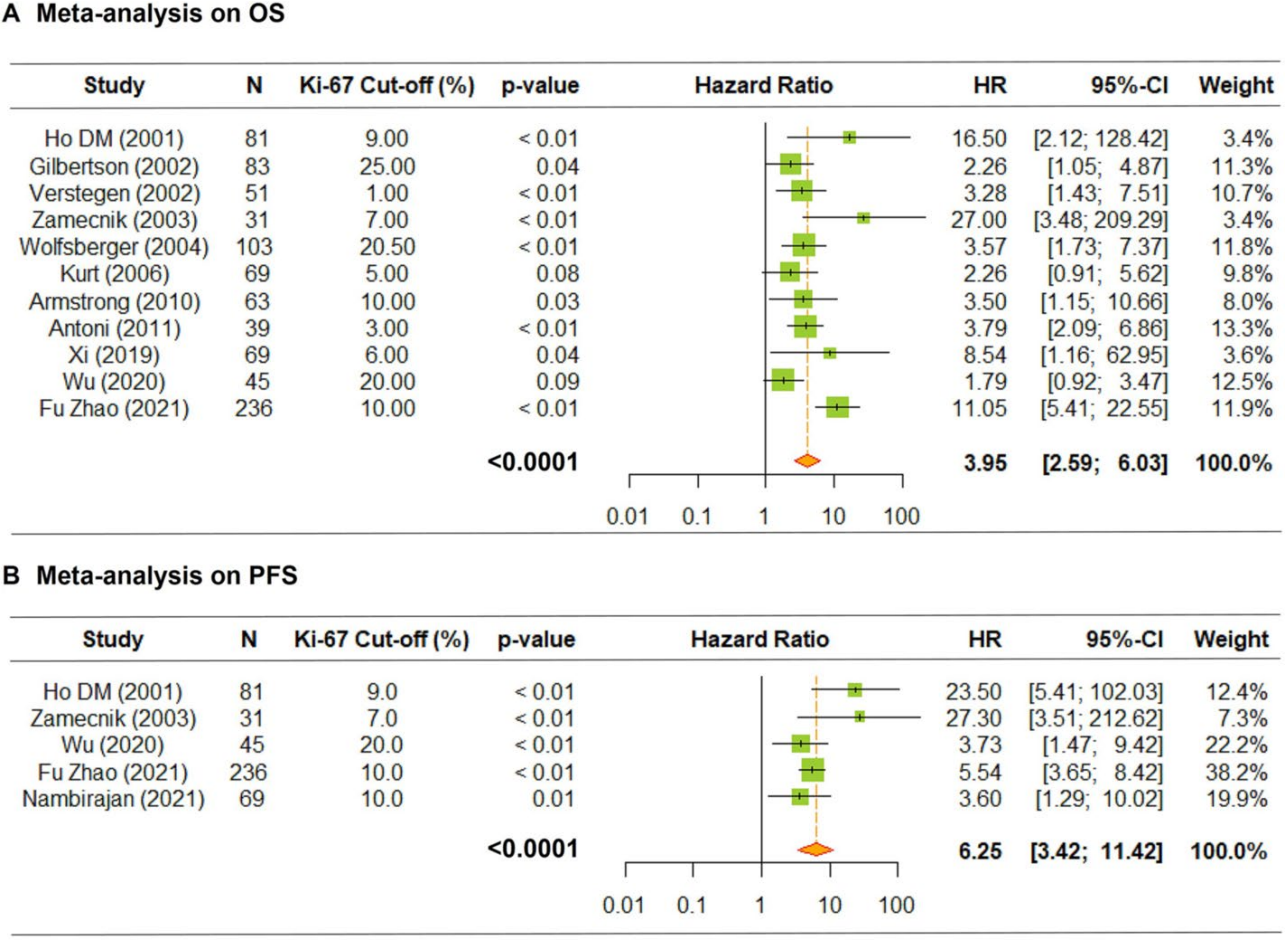
Meta-analysis about the prognostic value of Ki-67 index on OS (**A**) and PFS (**B**) of intracranial EPNs. Increased levels of Ki-67 were associated with poor survival rates (both *P* < 0.0001). The overall values were written in bold

A further meta-analysis of 11 publications [16, 18, 26–34], to identify the significance of Ki-67 labeling indices with the OS, showed that the overall HR was 3.95 (95% CI 2.59–6.03, *P* < 0.0001) indicating that increased levels of Ki-67 were associated with worse outcomes. The inter-study heterogeneity was negligible (Q = 22.8; *P* = 0.01) (Fig. 4A).

The overall HR of PFS of the five eligible studies was 6.25 (95% CI 3.42–11.42, *P* < 0.0001), suggesting a high association between increased Ki-67 indices and poor prognosis (Q = 7.41; *P* = 0.11) (Fig. 4B).

### Kaplan Meier survival analysis according to Ki-67 labeling indices, H3K27me3 loss, and EZHIP overexpression

**Fig. 5:**
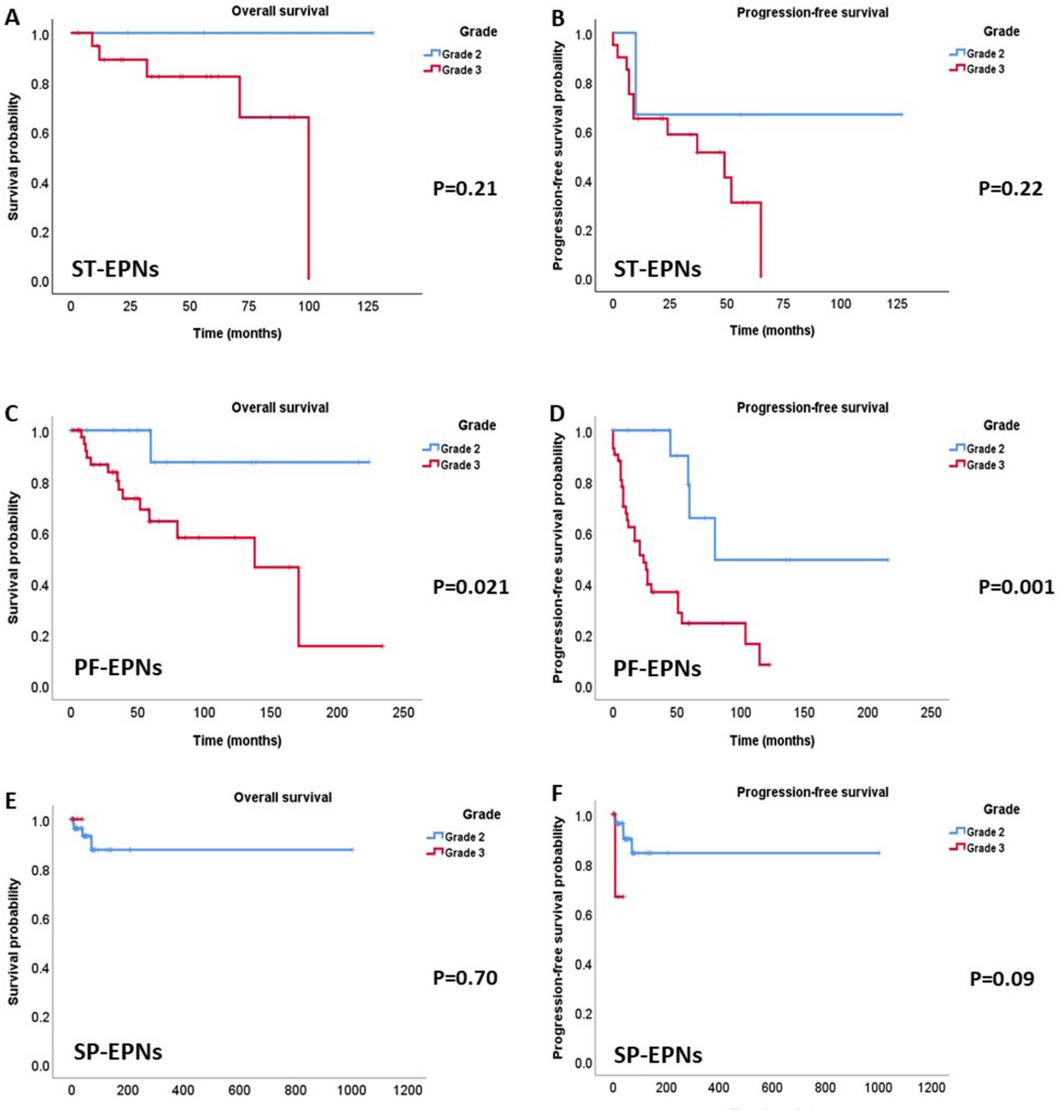
Kaplan–Meier survival analysis according to the grades in ST-, PF- and SP-EPNs. Grade 2 and grade 3 were designated by 7% of the Ki-67 cut-off. Grade 3 shows a significantly worse prognosis than grade 2, both in OS and PFS in PF-EPNs (**C**, **D**). However, the survival difference of ST- and SP-EPNs by the grade did not reach statistical significance (**A**, **B** and **E**, **F**)

The Kaplan–Meier survival analysis according to grade, the prognosis was worse in grade 3 than grade 2, but did not reach the statistical significance in ST-, and SP-EPNs (Fig. 5A, B and 5E, F) because the limitation of the number of WHO grade 2 ST-EPNs (6%) and WHO grade 3 SP-EPNs (8%). Both OS and PFS of PF-EPNs were strongly associated with the grades based on 7% Ki-67 (*P* = 0.021 and 0.001, respectively) (Fig. 5C, D).

Loss of H3K27me3 (*P* = 0.007) and overexpression of EZHIP (*P* = 0.002) were significantly associated with a poor prognosis. H3K27me3 and EZHIP were statistically significantly associated by Fisher’s exact test (*P* < 0.0001).

### A precise and easily applicable schematic diagnostic flow in clinical practice

**Fig. 6:**
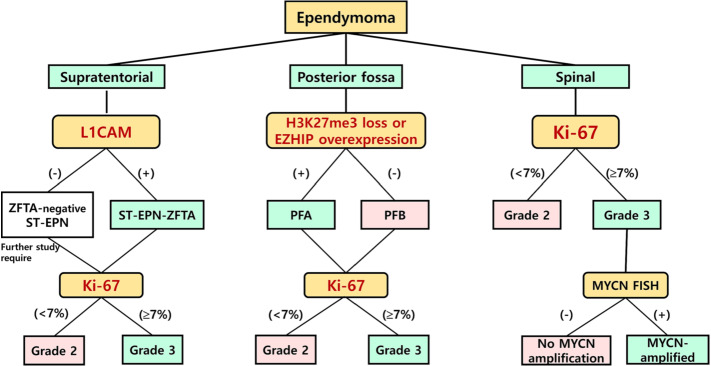
A schematic diagnostic flow that we propose. EPNs should be classified by the anatomic locations and the surrogate markers reflecting their genetic or epigenetic alteration. ST-EPN can be subdivided according to L1CAM-IHC. All L1CAM-positive EPNs can be considered as *ZFTA* fusion-positive EPNs. *ZFTA*-negative-ST-EPNs are heterogeneous tumors, which can be divided as WHO grade 2 or 3 according to the Ki-67 labeling index. PF-EPNs are subdivided according to H3K27me3 and EZHIP status. Both PFA and PFB can have both WHO grade 2 and 3 according to Ki-67 labeling indices. A Ki-67 index of 7% or more should be considered WHO grade 3. MYCN-FISH is not needed in WHO grade 2 SP-EPNs but essential in WHO grade 3 SP-EPNs

In our study, L1CAM and H3K27me3 (and/or EZHIP) IHC and Ki67 labeling indices were sufficient to classify intracranial EPNs, which could be both practical and cost-effective. H3K27me3 loss should be considered before EZHIP because the global reduction of H3K27me3 is the main mechanism of PFA-EPNs. For SP-EPNs, most had *NF2* gene deletion, monosomy 22, or rarely an *NF2* gene-splicing mutation but these genetic changes did not affect the patient's outcomes. The Ki-67 index was shown to be a powerful prognostic factor in multivariate analysis, and a lower than 7% cut-off was a good indicator for grades 2 EPNs. We proposed a schematic diagnostic flow of EPNs, which can be a useful, accurate, and cost-effective diagnosis in clinical and pathological practice (Fig. 6).

## Discussion

We successfully reclassified our 141 EPNs using three biomarkers of L1CAM, H3K27me3, and EZHIP, and Ki-67. Reclassified ependymomas showed a high correlation with pathological grade, patients’ age, and biological behavior. It was found that 70% of ST-EPN-*ZFTA* and almost all PFA-EPN occurred in children, but almost all PFB and 92% of low-grade SP-EPN were adults. Fusion genes were found in ST-EPN, except for one spinal EPN, which had *ZFTA-YAP1* fusion [36]. L1CAM was a practically excellent surrogate marker in predicting *ZFTA*-fusion, and H3K27me3 loss or EZHIP overexpression were excellent biomarkers to identify PFA-EPN. Since clear cell PFA-EPN showed only H3K27me3 loss, H3K27me3 loss may be a better biomarker than EZHIP for PFA-EPN diagnosis. In SP-EPNs, *NF2* mutation or deletion was the underlying genetic abnormality, but it did not affect biological behavior and did not have a role of surrogate marker for grading. 1q25 gain, monosomy 6, and CDKN2A/2B homozygous deletion were related to poor outcomes including multiple recurrences and extracranial metastases, which was similar to previous reports [37, 38].

The prognostic and predictive effects of histological parameters of EPN, including histological grade, are known to be controversial. According to Figarella-Branger et al., STR, loss of histological differentiation, high Ki-67 index (≥ 1%), and age younger than 4 years were associated with inferior PFS [39]. In one multivariate analysis by Horn et al., age (age < 3 vs. age ≥ 3), histologic grade (grade 2 vs. grade 3), and extent of resection affected OS and PFS [40].

In our study, the multivariate analysis showed that Ki-67 was the only independent prognostic factor in both PFS and OS, which can be supported by previous reports. Malgulwar et al.’s study revealed that L1CAM positivity and high Ki-67 labeling indices were associated with poor PFS, but the age and the extent of resection were not significant for survival [15].

In our study, the Ki-67 index (cut-off of 7%) was well correlated with the WHO grades and PFS of our series of EPNs in all anatomical locations. However, our cohort of EPNs did not show statistical significance in OS because our cohort has limitations in patients’ number and follow-up period. In addition, the patients in our cohort survived longer, the 5-year survival rates of higher grade (WHO grade 3) EPNs were 93.8%, 73.2%, and 93.8% in ST-EPN-*ZFTA*, PFA, and SP-EPN-*NF2*, respectively, and the 10-year survival rates were 81.3%, 70.7%, and 87.5%, respectively, suggesting that EPNs are chronic diseases, despite multiple local recurrence and distant metastases.

Although genetic and epigenetic subtypes were highly associated with the prognosis in intracranial EPNs, the previous studies reported that they do not necessarily determine the grades because there are some exceptional cases, such as grade 2 PFA with good prognosis, and grade 3 PFB and ST-EPN-*YAP1* with poor outcomes. [13, 41]

Reproducible cut-offs for Ki-67 labeling indices have been proposed in astrocytic and oligodendroglial tumors to predict the outcomes [42–45]. Similarly, our meta-analysis with 11 published papers showed that increased Ki-67 labeling indices were strongly correlated with poor OS and PFS of EPNs (both *P* < 0.0001).

Significant heterogeneity of staining techniques, antibody clones, and counting methods may affect cut-off. However, we used the same thickness of tissue sections and the same immunostaining method, and AperioSpectrum plus image analyzer to count all cases to maintain the same conditions and to minimize the inter-observer variability.

In our study, we found area-to-area differences of Ki-67 labeling indices, and grading based on the 7% cut-off in the hot spot area showed a strong association with genetic subtypes of EPNs and clinical outcomes. We conclude that a Ki-67 index of 7% is the most reliable cut-off for grading in all anatomical locations. Our cut-off is also supported by the study of Zamecnik et al. in which a Ki-67 labeling index more than and equal to 7% was associated with an inferior outcome in both OS and PFS of EPN [16].

In addition, our results showed that chromosome 1q25 gain and CDKN2A/2B loss were significant poor prognostic factors indicating a dismal prognosis, such as multiple recurrence or extracranial metastasis. The monosomy 6 and balanced chromosomal profile were the next worse prognostic factor, also indicating multiple recurrence and extracranial metastasis. However, multiple CNVs without the aforementioned CNVs were associated with favorable outcomes.

### Conclusion

Here, we reclassified EPNs according to the diagnostic criteria of cIMPACT-NOW update 7 and the  2021 WHO classification. From our study, we recommend a schematic diagnostic flow using three surrogate markers (L1CAM, H3K27me3, and EZHIP) and Ki-67 (Fig. 6), which may be a precise, easy and cost-effective diagnostic scheme for the management of patients.

## Supplementary Information

Below is the link to the electronic supplementary material.

Supplementary file1 (DOCX 25 KB) Supplementary Table 1. The primary antibodies used in this study. Supplementary Table 2. Copy number variations observed in 60 NGS-performed EPNs. Supplementary Table 3. Pathological, immunohistochemical, and genetic features of ST-, PF- and SP-EPNs

## Data Availability

The datasets used and/or analyzed during the current study are available from the corresponding author on reasonable request.

## Abbreviations

EPN: Ependymoma
CNS: Central nervous system
ST: Supratentorial
PF: Posterior fossa
SP: Spinal
WHO: World Health Organization
RT: Radiation therapy
CT: Chemotherapy
CCRT: Concurrent chemo-radiotherapy
H3K27me3: Trimethylation of lysine 27 of histone H3
CIMP: CpG island methylator phenotype
NF2: Neurofibromatosis type 2
OS: Overall survival
PFS: Progression-free survival
FISH: Fluorescent in situ hybridization
IHC: Immunohistochemistry
NGS: Next-generation sequencing
WES: Whole-exome sequencing
FFPE: Formalin-fixed paraffin-embedded
SNV: Single nucleotide variant
CNV: Copy number variation
NOS: Not otherwise specified
GTR: Gross total resection
STR: Subtotal resection
HR: Hazard ratio
95% CI: 95% Confidence interval
