# Supplementary material for: Molecular subtyping of ependymoma and prognostic impact of Ki-67
Source: Brain Tumor Pathol. 2021 Nov 23;39(1):1–13. doi: 10.1007/s10014-021-00417-y (PMC8752536; doi:10.1007/s10014-021-00417-y)
Supplement: Supplementary file 1 — Supplementary file1 (DOCX 25 KB) Supplementary Table 1. The primary antibodies used in this study. Supplementary Table 2. Copy number variations observed in 60 NGS-performed EPNs. Supplementary Table 3. Pathological, immunohistochemical, and genetic features of ST-, PF- and SP-EPNs [file 10014_2021_417_MOESM1_ESM.docx]

**Supplementary Table 1. The primary antibodies used in this study**

| Antibody | Dilution | Antigen retrieval | Clone | Source |
| --- | --- | --- | --- | --- |
| L1CAM | 1:10,000 | Ventana CC1 at 100^o^C | UJ127 (monoclonal) | Sigma-aldrich, Saint Louis, USA |
| NFkB p65/RELA | 1:1,000 | Ventana CC1 at 100^o^C | D14E12 (monoclonal) | Cell Signaling, Boston, USA |
| YAP1 | 1:300 | Ventana CC1 at 100^o^C | SC-101199 (monoclonal) | Santa Cruz, Texas, USA |
| H3K27me3 | 1:100 | Ventana CC1 at 100^o^C | C36B11 (monoclonal) | Cell Signaling, Boston, USA |
| EZHIP (CXorf67) | 1:300 | Ventana CC1 at 100^o^C | HPA004003 (polyclonal) | Atlas, Stockholm, Sweden |
| GFAP | 1:200 | Ventana CC1 at 100^o^C | 6F2 (monoclonal) | DAKO, Glostrup, Denmark |
| K27M | 1:500 | Ventana CC1 at 100^o^C | ABE419 (polyclonal) | Milipore, Temecula, USA |
| Ki67 | 1:100 | Ventana CC1 at 100^o^C | M7240 (monoclonal) | DAKO, Glostrup, Denmark |
| P16 | RTU | Ventana CC1 at 100^o^C | E6H4 (monoclonal) | Ventana, Export, USA |
| P53 | 1:100 | Ventana CC1 at 100^o^C | DO7 (monoclonal) | DAKO, Glostrup, Denmark |
| pHH3 | 1:100 | Ventana CC1 at 100^o^C | 369A-15 (polyclonal) | Cell Marque, Rocklin, USA |

:, GFAP, glial fibrillary acidic protein; K27M, antibody for Histon Lys27Met; pHH3, phosphorylated Histone H3, RTU: ready to use

**Supplementary Table 2. Copy number variations observed in 60 NGS-performed EPNs**

| CNV  N=60 | ST-EPNs (n=16) | | PF-EPNs (n=23) | | SP-EPNs (n=21) | |
| --- | --- | --- | --- | --- | --- | --- |
|  | Grade 2 (n=1) | Grade 3 (n=15) | Grade 2 (n=4) | Grade 3 (n=19) | Grade 2 (n=16) | Grade 3 (n=5) |
| 1q25 gain | - | 3 | - | 5 | - | - |
| CDKN2A/2B deletion | - | 2 (Homozygous) | - | 2 (Homozygous)  2 (Hemizygous) | - | - |
| Chr 6 monosomy | - | 1 | 1 | 2* | 1 | - |
| Chr 11 monosomy | - | 1 | - | - | - | - |
| Balanced | 1 | 5 | - | 3 | 1 | 2 |
| NF2 splicing mutation | - | - | - | - | - | 2 |
| NF2 gene deletion | - | - | 1 (Homozygous) | - | 7 (6 hemizygous, 1 homozygous) | 1 (hemizygous) |
| Chr 22 monosomy | - | 2 | - | 2* | 6 | - |
| Others | - | 1 (1p deletion) | 2 (Multiple CNV) | 1 (Chr 10 monosomy)  1 (Chr 7 monosomy)  1 (Chr 19q gain)  1 (Chr 1 & Chr 9 monosomy) | 1 (Multiple CNV) | - |

*: One PFA-EPN had both chromosomes 6 and 22 monosomy; CNV: copy number variation

**Supplementary Table 3. Pathological, immunohistochemial and genetic features of ST-, PF- and SP-EPNs.**

| Parameters Subtype | ST-EPN-*ZFTA*  (n=16) | ST-EPN-*YAP1*  (n=1) | IHC results | PFA  (n=41) | PFB  (n=17) | Genetic  alteration | SP-EPN Grade 2  (n=61) | SP-EPN Grade 3  (n=5) |
| --- | --- | --- | --- | --- | --- | --- | --- | --- |
| Coventional | 16 | 1 |  | 38 | 14 |  | 49 | 5 |
| papillary | - | - |  | 2 | 3 |  | 2 | - |
| clear cell | - | - |  | 1 | - |  | 1 | - |
| tanycytic | - | - |  | - | - |  | 9 | - |
| Microvascular proliferation | 16 | - |  | 25 | 1 |  | 0 | 5 |
| Mitoses/10 HPF: median (range) | 9.5 (3-35) | 1 |  | 23 (2-85) | 2 (0-45) |  | 1 (0-4) | 10 (7-30) |
| Necrosis | 13 | 1 |  | 30 | 7 |  | 7 | 3 |
| Calcification | 9 | 1 |  | 25 | 4 |  | 11 | 1 |
| L1CAM positive | 16 | - | H3K27me3 loss | 41 | 0 |  |  |  |
| NFkb positive | 14 | - | EZHIP overexpression | 40 | 0 |  |  |  |
| YAP1 positive | 10 | 1 | K27M positive | 0 | 0 |  |  |  |
| Ki-67: Median (range) | 35.8% (9.0~87.4) | 4.6% |  | 31% (2.5~77.1) | 3.2% (0.9~26.6) |  | 1.7% (0.1~6.2) | 30% (19~58.6) |
| Grade 2 | 0 | 1 |  | 3 | 13 |  |  |  |
| Grade 3 | 16 | - |  | 38 | 4 |  |  |  |
| *ZFTA-RELA* | 15 | - |  |  |  | *NF2* alteration | 13 | 3 |
| *ZFTA-MAML2* | 1 | - |  |  |  | *ZFTA-YAP1* | - | 1 |
| *YAP1-MAMLD1* | 0 | 1 |  |  |  |  |  |  |

IHC: immunohistochemistry
